# A Guide to the Patient-Centric Use of Vedolizumab for Crohn’s Disease

**DOI:** 10.1093/ibd/izaf072

**Published:** 2025-06-13

**Authors:** David T Rubin, Ailsa L Hart

**Affiliations:** University of Chicago Medicine Inflammatory Bowel Disease Center, Chicago, IL, USA; St Mark’s National Bowel Hospital, London, UK

**Keywords:** biologics, clinical guidelines, Crohn’s disease, vedolizumab

## Abstract

Crohn’s disease (CD) is a chronic, progressive, and debilitating disease characterized by inflammation of the gastrointestinal tract. It can have a significant impact on quality of life if not adequately controlled by appropriate treatment. In recent years, disease management strategies for CD have evolved from a focus on treating symptoms toward the additional goal of early treatment to modify disease progression and prevent bowel damage. Advanced therapies, including biologics, are a key part of the modern medical treatment paradigm for CD, but their introduction has made the treatment landscape more complex. Knowing which advanced therapy to use, when, and for which patient is challenging for health care providers. The α_4_β_7_ integrin inhibitor vedolizumab has demonstrated effectiveness, tolerability, and an acceptable long-term safety profile in patients with CD. In this review, we discuss the use of vedolizumab in the management of CD to help clinicians make evidence-based treatment decisions and maximize benefits for their patients. We summarize the medical management of CD, the unique gut-selective mechanism of action of vedolizumab, and the current clinical guidelines for using vedolizumab in patients with CD. We detail the ability of vedolizumab to achieve mucosal healing and alter disease progression and present the evidence that supports the use of vedolizumab as a first-line biologic early in the disease course in patients with CD. Finally, we address some of the perceived drawbacks of using vedolizumab in CD, including concerns about slow onset of action, impact of disease location, and exacerbation of extraintestinal manifestations.

## Introduction

Crohn’s disease (CD) is characterized by relapsing and remitting or chronic progressive inflammation that can affect the entire gastrointestinal tract, but most frequently impacts the terminal ileum and proximal colon.^1^ It can be associated with irreversible structural changes including fibrosis and ulceration that lead to strictures, abscesses, and fistulae affecting the full thickness of the bowel wall and adjacent structures.^1,2^ The underlying causes are multifactorial, involving genetic factors; the patient’s microbiota and intestinal immune system; and environmental factors, including smoking status and diet.^1,3^

The clinical course and symptoms of CD can vary significantly^4–6^ but patients typically experience abdominal pain, loss of appetite, weight loss, diarrhea, rectal bleeding, and fatigue.^1,3,7^ Extraintestinal manifestations have been estimated to occur in 6% to 47% of patients with inflammatory bowel disease (IBD)^8^ and include skin disorders (eg, pyoderma gangrenosum and erythema nodosum), rheumatic disorders (eg, peripheral arthritis), and ophthalmic conditions (eg, anterior uveitis).^9^ Although patients may present with mild disease, the progressive nature of CD means that a high proportion will go on to develop serious complications if the disease is not adequately treated.^6^

CD is associated with considerable damage to the intestine and, historically, rates of surgery are high. Surgery for CD is not always curative, and postsurgical recurrence rates remain high; 5 years after surgery, endoscopic recurrence may be as high as 89%.^10^ Moreover, surgery is associated with potentially serious complications including anastomotic leak, intra-abdominal bleeding, intestinal obstruction, and infection, and can, rarely, result in death.^11^ A mortality rate of 0.6% has been estimated for elective surgery in patients with CD, and this rises to 3.6% when emergency surgery is needed.^11^

### Management of CD

Curative therapy is not yet available for CD. Previously, medical management involved a step-up approach that focused on treating symptoms of the disease. Patients would typically be started on steroids, aminosalicylates, or thiopurine immunomodulators and would then be escalated to more effective therapies as each line of treatment failed. However, this approach had minimal effect on disease progression, meaning many patients continued to have poorly controlled disease and ultimately required surgery.^1^

The ultimate goal of modern medical therapy in CD is to alter the natural history of the disease to prevent, or significantly delay, the progression that leads to poor outcomes. With the evolution of disease management strategies, the emergence of new treatment targets, and the introduction of advanced therapies, the treatment landscape has become increasingly complex.

#### Emerging Therapeutic Targets in CD

Clinical symptoms are inconsistent indicators of mucosal inflammation, and significant inflammation may persist in patients who have complete clinical remission^12^; therefore, monitoring objective signs of inflammation or disease biomarkers is seen as important to achieving optimal treatment outcomes.^13–15^

In 2015, the Selecting Therapeutic Targets in Inflammatory Bowel Disease (STRIDE) initiative of the International Organization for the Study of Inflammatory Bowel Diseases (IOIBD) recommended several treatment targets for monitoring disease progression.^13^ The updated initiative STRIDE-II recognizes endoscopic response (defined as a > 50% reduction in Simple Endoscopic Score for Crohn’s Disease [SES-CD] or Crohn’s Disease Endoscopic Index of Severity [CDEIS]) as an important short-term target for treatment of CD and endoscopic healing (defined as SES‑CD < 3 or the absence of ulcerations) as an important long-term target. For patients who do not have endoscopic healing after receiving a specific treatment course, it is recommended that a switch should be considered.^14^ STRIDE-II also endorses the use of normalization of C‑reactive protein (CRP) or fecal calprotectin (FCP) levels as an intermediate biomarker target for patients with CD, with the recommendation to consider a treatment switch for patients when CRP level or FCP level cannot be reduced to values under the upper limit of normal or to 100 µg/g to 250 µg/g, respectively.^14^

Deep remission is a treatment concept that initially involved combining clinical remission (a Crohn’s Disease Activity Index [CDAI] < 150) with endoscopic evidence of mucosal healing (complete absence of ulceration) as a composite endpoint.^13^ More recently, it has been recommended that deep remission could also include transmural healing,^14^ with the possibility that future definitions may also include assessment of biomarker normalization (eg, CRP or FCP level).^16^

Endoscopic measurement of mucosal healing remains the gold standard for objective assessment of inflammation in CD and is a strong predictor of long-term positive outcomes, including steroid-free remission and lower rates of surgery.^14,17–19^ However, CD is characterized by transmural inflammation that may persist even when full mucosal healing occurs.^20^ Consequently, the use of cross-sectional diagnostic approaches including intestinal ultrasound, computed tomography enterography, or magnetic resonance enterography (MRE) to measure transmural healing across the entire thickness of the bowel has emerged as another potential therapeutic goal.^20^ In STRIDE-II, transmural healing is recommended as an adjunct to endoscopic remission to represent a deeper level of healing in patients with CD.^14^ Several studies have subsequently demonstrated the association of transmural healing with improved patient outcomes versus endoscopic mucosal healing, including higher rates of steroid-free remission,^20,21^ lower risk of bowel damage,^22^ and lowered rates of hospitalization or surgery.^20,21^ However, rates of transmural healing in CD with different advanced therapies are not yet clear and are the subject of ongoing research (eg, in the ongoing VECTORS study [NCT06257706]).

#### Treating the Right Patient at the Right Time

The recognition that earlier intervention with biologics may prevent disease progression, reduce bowel damage and long-term complications, and improve patient health-related quality of life (HRQoL) has been a pivotal shift in disease management.^13,14,23^ For example, in the open-label, randomized PROFILE study, top-down treatment with infliximab plus an immunomodulator achieved better outcomes at 1 year than accelerated step-up treatment with conventional therapies in patients with newly diagnosed CD.^24^ Overall, early biologic treatment, defined as the initiation of biologics less than 2 years after CD diagnosis or before treatment with oral immunosuppressants, is associated with improved clinical outcomes in prospective clinical trials and in real-world clinical settings.^25^ A meta-analysis found early biologic treatment was associated with higher rates of clinical remission than late biologic treatment or conventional management (odds ratio [OR], 2.10; 95% CI, 1.69 to 2.60, *P* < .00001).^25^ However, early use of biologic therapy in all patients may not be economically viable or desirable in those with an indolent disease course in whom overtreatment and unnecessary biologic exposure could lead to increased risks of adverse effects.^1^

Stratifying patients to identify those who are most likely to have an aggressive disease course, and would benefit from early intervention with biologics, has become a key goal of disease management.^4,14^ Prognostic risk factors for disease progression and complicated disease include young age at diagnosis, ileal or ileocolonic disease, extensive small bowel involvement, severe upper gastrointestinal disease, early stricturing or penetrating disease, rectal disease, perianal lesions, and smoking.^1,26,27^ As well as prognostic disease risk factors, the identification of predictive factors for response to advanced therapies may also help to guide treatment decisions. For example, tools have been developed that can predict subgroups of patients who may have better outcomes with biologic treatment.^28–30^

#### Vedolizumab: A Gut-Selective Targeted IBD Therapy

Several targeted IBD therapies have been approved for use or are under investigation for use in patients with CD, including anti-tumor necrosis factor α (anti-TNFα) antagonists (eg, infliximab, adalimumab, and certolizumab pegol), which were the first to receive approval for treatment of CD, interleukin antagonists (eg, ustekinumab and risankizumab), and Janus kinase inhibitors (eg, upadacitinib).^1,3^ However, since its approval by the European Medicines Agency and the US Food and Drug Administration in 2014, vedolizumab has been the only gut-selective anti-lymphocyte trafficking therapy indicated for use in CD.^31,32^ It is indicated for the treatment of patients with moderately to severely active CD who have had an inadequate response with, lost response to, or were intolerant to either conventional therapy or an anti-TNFα treatment.^31,32^

**Figure 1.**
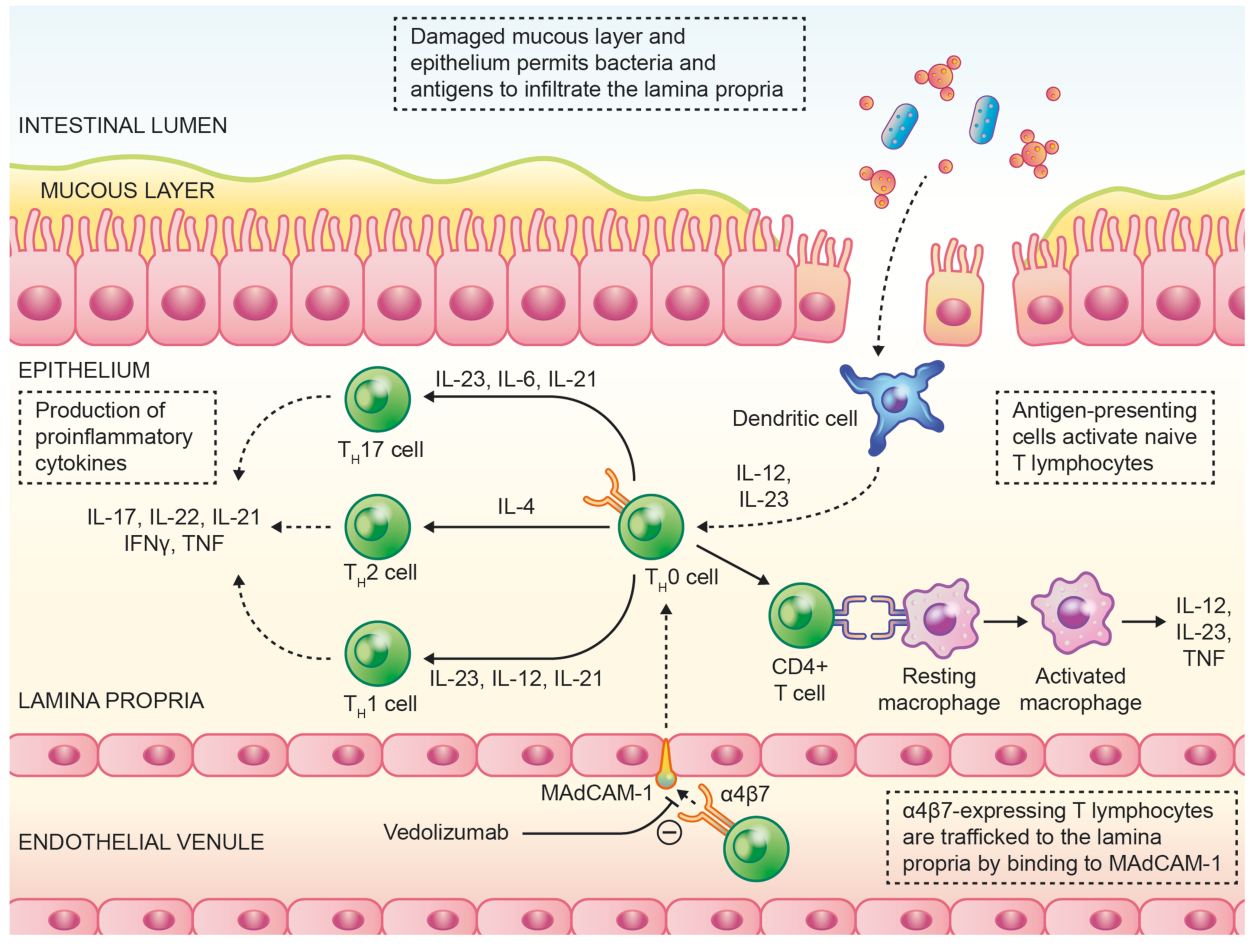
Dysregulated lymphocyte trafficking in CD and the mechanisms of action of vedolizumab. Abbreviations: CD, Crohn’s disease; IFN, interferon; IL, interleukin; MAdCAM-1, mucosal vascular addressin cell adhesion molecule 1; TNF, tumor necrosis factor.

The pathways involved in lymphocyte trafficking, CD pathology, and the mechanism of action of vedolizumab have been described in detail by others.^33,34^ Vedolizumab binds preferentially to the α_4_β_7_ integrin heterodimer expressed on T lymphocytes and prevents its interaction with mucosal vascular addressin cell adhesion molecule 1 (MAdCAM-1) on gut endothelial cells (**Figure 1**).^31,32,35^ The interaction between α_4_β_7_ integrin and MAdCAM-1 is important for the controlled trafficking of gut-homing T lymphocytes to the intestinal mucosa which is critical for healthy gut immunosurveillance.

Unlike other anti-integrins, such as natalizumab (which inhibits both the binding of α_4_β_7_ to MAdCAM-1 and α_4_β_1_ integrin to VCAM-1) or etrolizumab (which binds to the β_7_ subunit of both α_4_β_7_ and αEβ_7_), vedolizumab inhibits only the binding of α_4_β_7_ integrin to MAdCAM-1.^33^ This highly selective binding endows vedolizumab with a gut-selective mechanism of action, because both MAdCAM-1 and α_4_β_7_ integrin are preferentially expressed in gut-associated lymphoid tissue, with low expression in extraintestinal tissues.^36^ By blocking α_4_β_7_ integrin, vedolizumab inhibits the trafficking of several different immune cells to the gut, particularly T lymphocytes, to reduce the excretion of proinflammatory cytokines and to reduce the inflammatory response associated with CD.^33^ As discussed later in this review, the gut selectivity of vedolizumab has translated into a long-term safety and tolerability profile that favors the use of this treatment over other biologics in some patients.

### Vedolizumab Is Effective in the Treatment of CD

#### Clinical outcomes

A summary of the evidence showing that vedolizumab can produce positive clinical outcomes is presented in **Table 1**. In the pivotal phase 3 GEMINI 2 trial, vedolizumab administered intravenously (IV) demonstrated efficacy for induction and maintenance of clinical remission (CDAI ≤ 150) in patients with moderate to severe CD. At week 6, 14.5% of patients who received induction doses of vedolizumab 300 mg IV at weeks 0 and 2 were in clinical remission compared with 6.8% of those who received placebo (*P* = .02). In the maintenance phase of the study, patients who had a clinical response (≥ 70-point reduction in CDAI from baseline) at week 6 were randomized to receive vedolizumab 300 mg IV, either once every 8 weeks (Q8W) or once every 4 weeks (Q4W), or placebo. At week 52, 39.0% and 36.4% of patients receiving vedolizumab Q8W and Q4W, respectively, were in clinical remission (the primary endpoint) compared with 21.6% who received placebo (*P* < .001 and *P* = .004, respectively). In patients who had a clinical response at week 6, 43.5% who received vedolizumab Q8W and 45.5% who received vedolizumab Q4W had a clinical response at week 52, compared with 30.1% who received placebo (*P* = .01 and *P* = .005, respectively). A post hoc analysis of data from GEMINI 2 revealed that patients treated with vedolizumab who had moderate CD (CDAI ≤ 330) had a greater reduction in abdominal pain and stool frequency by week 6 and higher rates of clinical response and remission at week 52 than those with severe disease (CDAI > 330).^63^ Corticosteroid-free remission at week 52 was observed in 31.7% and 28.8% of patients who received vedolizumab Q8W and Q4W, respectively, compared with 15.9% of those who received placebo (*P* = .02 and *P* = .04, respectively).^37^ In the GEMINI 3 study of vedolizumab induction therapy, in the overall patient group, which comprised patients who had prior anti-TNFα treatment failure and patients who were anti‑TNFα treatment-naive, vedolizumab produced higher rates of clinical response (CDAI decrease ≥ 100-points) and clinical remission (CDAI ≤ 150) at weeks 6 and 10 than did placebo.^38^

**Table 1.** Clinical outcomes in clinical trials and real-world studies of vedolizumab in patients with CD.

Study name	Endpoint/Outcome (definition)	Time point	Rate
**Clinical trials**			
GEMINI 2^37^	Clinical response (CDAI ≥ 100-point decrease)	Week 6	VDZ: 31.4%; PBO: 25.7%
		Week 52	VDZ^a^: 43.5%; VDZ^b^: 45.5%; PBO: 30.1%
	Clinical remission (CDAI ≤ 150)	Week 6	VDZ: 14.5%; PBO: 6.8%
		Week 52	VDZ^a^: 39.0%; VDZ^b^: 36.4%; PBO: 21.6%
GEMINI 3^38^	Clinical response (CDAI ≥ 100-point decrease)^c^	Week 6	VDZ: 39.2%; PBO 22.7%
		Week 10	VDZ: 47.8%; PBO 24.2%
	Clinical remission (CDAI ≤ 150)^c^	Week 6	VDZ: 19.1%; PBO 12.1%
		Week 10	VDZ: 28.7%; PBO 13.0%
GEMINI LTS^39,40^	Clinical response (HBI ≥ 3-point decrease)	Week 104	78.0%^b^^,^^d^
		Week 152	47.0%^b^^,^^d^
		Week 400	33.1%^b^
	Clinical remission (HBI ≤ 4)	Week 104	69.0%^b^^,^^d^
		Week 152	43.0%^b^^,^^d^
		Week 400	28.1%^b^
VISIBLE 2^41^	Clinical response (CDAI ≥ 70-point decrease)	Week 6	64%
	Enhanced clinical response (CDAI ≥ 100-point decrease)	Week 52	VDZ^e^: 52.0%; PBO: 44.8%
	Clinical remission (CDAI ≤ 150)	Week 52	VDZ^e^: 48.0%; PBO: 34.3%
VERSIFY^42^	Clinical response (CDAI ≥ 100-point decrease)	Week 10	54.5%
		Week 26	60.4%
		Week 52	58.9%^f^
	Clinical remission (CDAI ≤ 150)	Week 10	35.6%
		Week 26	41.6%
		Week 52	50.0%
LOVE-CD^43^	Clinical response (CDAI > 70-point decrease)	Week 26	38%
		Week 52	35%
	Corticosteroid-free clinical remission (CDAI < 150 and no corticosteroid use)	Week 26	29%
		Week 52	31%
**Real-world data**			
OBSERV-IBD^44–46^	Clinical response (HBI ≥ 3-point decrease)	Week 6	56.6%
		Week 14	63.6%
		Week 54	47.4%
		Week 162	21.1%
	Clinical remission (HBI ≤ 4)	Week 6	31.2%
		Week 14	36.4%
		Week 54	30.0%
		Week 162	19.9%
US VICTORY consortium registry analysis^47^	Clinical response (physician global assessment of ≥ 50% reduction in CD-related symptom activity and/or severity)	Week 6	40.6%
		Month 6	32.0%
		Month 12	58.0%
	Clinical remission (physician global assessment complete resolution in all CD-related symptoms)	Week 6	10.9%
		Month 6	18.0%
		Month 12	35.0%
Schreiber 2018^48^	Clinical response (HBI ≥ 3-point decrease; improvement in HBI; CDAI > 100-point decrease; CRP > 50% decrease; CD-related symptom activity or severity based on PGA ≥ 50% decrease)	Week 6	56%
		Week 14	58%
		Month 12	40%
	Clinical remission (HBI ≤ 4; HBI < 5; CDAI < 150; CRP < 5 mg/L; complete resolution of all CD-related symptoms; any reduction in HBI score from before VDZ initiation to after 14 weeks of therapy)	Week 6	24%
		Week 14	30%
		Month 12	30%
Baumgart 2016^49^	Clinical response (HBI ≥ 3-point decrease)	Week 6	66.0%
		Week 14	60.8%
	Clinical remission (HBI ≤ 4)	Week 6	15.5%
		Week 14	23.7%
Abramowitz 2016^50^	Clinical response (HBI ≥ 3-point decrease)	Week 10 or 14	60%
	Clinical remission (HBI ≤ 4)	Week 10 or 14	37%
Chaparro 2016^51^	Clinical response (definition not provided)	Week 14	62.0%
	Clinical remission (definition not provided)	Week 14	14.3%
Eriksson 2017^52^	Clinical response (P-HBI ≥ 3-point decrease)	Week 12	44%
		Week 52	53%
		Last follow‑up (median 17 months)	51%
	Clinical remission (P-HBI < 5)	Week 12	49%
		Week 52	60%
		Last follow‑up (median 17 months)	54%
Glover 2015^53^	Clinical remission (HBI < 5)	Week 14	17.9%
Mankongpaisarnrung 2016^54^	Clinical remission/response (definition not provided)	Weeks 8-12	27.3%
Shelton 2015^55^	Clinical response (HBI ≥ 3-point decrease)	Week 14	48.9%
	Clinical remission (HBI ≤ 4)	Week 14	23.9%
Christensen 2018^56^	Clinical response (HBI ≥ 3-point decrease)	Week 14	58%
		Week 30	73%
		Week 52	56%
	Clinical remission (HBI ≤ 4)	Week 14	38%
		Week 30	62%
		Week 52	51%
Kopylov 2018^57^	Clinical response (improvement of ≥ 1 severity score category)	Week 14	82.0%
		Last follow-up (median 30 weeks)	77.1%
	Clinical remission (HBI ≤ 4, CDAI < 150)	Week 14	64.0%
		Last follow-up (median 30 weeks)	68.6%
Plevris 2019^58^	Clinical remission (HBI < 5 and complete tapering of steroids [PGA used in patients with stoma])	Month 3	21.9%
		Month 6	39.9%
		Month 12	58.4%
Faleck 2019^59^	Clinical remission (physician assessment as complete resolution of all CD-related symptoms)	Month 6	Early-stage CD^g^: 38%; late-stage CD^h^: 23%
Perin 2019^60^	Clinical response (HBI ≥ 3-point decrease)	Week 12	48.97%
		Week 26	66.60%
		Week 52	50.00%
	Clinical remission (HBI ≤ 4)	Week 12	42.89%
		Week 26	61.90%
		Week 52	46.15%
Hanžel 2019^61^	Clinical remission (resolution of abdominal pain and altered bowel habit)	Week 54	57%
Reinglas 2020^62^	Clinical remission (overall HBI score < 5 and resolution of fistula drainage)	Month 3	9.1%
		Month 6	26.7%
		Month 12	29.2%

^a^VDZ 300 mg IV Q8W.

^b^VDZ 300 mg IV Q4W.

^c^Overall group including patients with prior anti-TNFα treatment failure and anti-TNFα treatment-naive patients.

^d^Missing data imputed as treatment failure.

^e^VDZ 108 mg subcutaneously every 2 weeks.

^f^Data from sub-study population at week 52 (n = 56 patients).

^g^Early-stage CD was defined as disease duration ≤ 2 years.

^h^Late-stage CD was defined as disease duration > 2 years. VDZ 300 mg was administered IV Q8W after induction unless otherwise stated.

Abbreviations: Anti-TNFα, anti-tumor necrosis factor α; CD, Crohn’s disease; CDAI, Crohn’s Disease Activity Index; CRP, C-reactive protein; GEMINI LTS, GEMINI long-term safety; HBI, Harvey-Bradshaw Index; IBD, inflammatory bowel disease; IV, intravenously; PBO, placebo; PGA, Physician’s Global Assessment; P-HBI, patient-based HBI; Q4W, every 4 weeks; Q8W, every 8 weeks; VDZ, vedolizumab.

The open-label GEMINI long-term safety (GEMINI LTS) study demonstrated that the clinical benefits of vedolizumab were sustained for up to 400 weeks after treatment initiation. In patients who had a clinical response (≥ 3-point decrease in Harvey-Bradshaw Index [HBI] from baseline) at week 6 and had later data available, 83% (*n* = 100/120) and 89% (*n* = 62/70) had clinical remission (HBI ≤ 4) after 104 weeks and 152 weeks of continuous vedolizumab treatment, respectively. Clinical response was reported in 94% and 97% of patients at week 104 and week 152, respectively. When accounting for missing data by imputing patients without data as treatment failures, at week 104 and week 152, respectively, clinical response rates were 78% and 47%, and clinical remission rates were 69% and 43%.^39^ After 400 weeks of treatment, 28% of patients were in clinical remission and 33% of patients had a clinical response.^40^

The VISIBLE 2 trial evaluated the efficacy of maintenance treatment with vedolizumab administered subcutaneously (SC) in patients with moderately to severely active CD who achieved a clinical response (≥ 70-point decrease in CDAI score from baseline) to standard induction treatment with vedolizumab IV at week 6. In patients who received vedolizumab 108 mg every 2 weeks SC from week 6 (*n* = 275), 48.0% were in clinical remission (primary endpoint; CDAI ≤ 150) at week 52, a difference of 13.7% (95% CI, 3.8 to 23.7; *P* = .008) compared with placebo (34.3%).^41^ A subgroup analysis revealed that patients with moderate disease severity (CDAI ≤ 330) at baseline had a greater difference in clinical remission between those who received vedolizumab and those who received placebo (Δ: 17.3%; 95% CI, 4.3 to 30.3) than those with severe disease (CDAI > 330) at baseline (Δ: 8.8%; 95% CI, − 6.7 to 24.2). The proportion of patients who were in clinical remission at week 52 was similar to those who received vedolizumab IV in the GEMINI 2 trial (Δ vedolizumab versus placebo: Q8W IV, 17.4%; Q4W IV, 14.8%).^37^ In VISIBLE 2, corticosteroid-free remission at week 52 was observed in 45.3% of patients who received vedolizumab compared with 18.2% of those who received placebo.^41^

There is extensive real-world evidence showing that outcomes in patients with CD are similar or better in clinical practice than in clinical trials (**Table 1**). In the OBSERV‑IBD study, a retrospective analysis including 173 patients with CD who received premarket-approval treatment with vedolizumab across 41 centers in France, 31% of patients were in clinical remission at week 6, of whom 19% had corticosteroid-free clinical remission. By week 14, rates of clinical remission and corticosteroid-free clinical remission were 36% and 31%, respectively. At weeks 6 and 14, respectively, 57% and 64% of patients had a clinical response to vedolizumab induction treatment.^44^ At week 54, 30.0% of patients were in clinical remission and 27.2% were in corticosteroid-free clinical remission.^45^ At 162 weeks after treatment initiation, the rate for both outcomes was 19.9%.^46^

Similarly, in a retrospective cohort study on the real-world effectiveness of vedolizumab that was conducted in the US by the VICTORY consortium and included 212 patients with moderate to severe CD, 10.9% of patients, 91% of whom had prior anti-TNFα treatment exposure, were in clinical remission at week 6.^47^ Following 12 and 18 months of maintenance treatment with vedolizumab, respectively, 35% and 54% of patients were in clinical remission.^47^ Patients who had moderate severity disease at baseline were more likely to achieve clinical remission (hazard ratio [HR], 0.54; 95% CI, 0.31 to 0.95) than those with severe disease.^47^

In a systematic review and meta-analysis of the real-world safety and effectiveness of vedolizumab in patients with CD, the pooled clinical remission rates at 6 weeks, 14 weeks, 6 months, and 12 months were 24% (95% CI, 20% to 27%; *n* = 598 patients assessed), 30% (95% CI, 25% to 34%; *n* = 756), 26% (95% CI, 19% to 35%; *n* = 505), and 30% (95% CI, 20% to 42%; *n* = 595), respectively.^48^ Combined real-world clinical response rates were 56% at week 6 and 40% at 12 months in this analysis.^48^ In a separate meta-analysis that included real-world studies of patients treated with vedolizumab, 49% and 32% of patients, respectively, had a clinical response and were in clinical remission at week 14 (*n* = 621 patients in 5 studies); rates at week 52 were 45% and 32%, respectively (*n* = 347 patients in 3 studies).^64^

#### Prevention of Postsurgical Recurrence

Despite improvements in medical therapy, many patients with CD still need to undergo surgery. The most common surgical intervention, ileocolonic resection, is associated with a high rate of postsurgical CD recurrence.^65^ Observational studies and case series have reported that vedolizumab may be effective in reducing postsurgical recurrence rates.^65,66^ The prospective, placebo-controlled, randomized REPREVIO study is investigating the preventative impact of initiating vedolizumab up to 4 weeks after surgery in patients at 12 sites in the Netherlands, France, Italy, and Spain. At week 26 of the study, patients in the vedolizumab treatment group (*n* = 43) had a 77.8% probability (95% CI, 66.4 to 86.3, *P* < .0001) of having a lower modified Rutgeerts score than patients in the placebo group (*n* = 37). The proportion of patients with severe endoscopic recurrence (modified Rutgeerts score ≥ i2b) was lower in vedolizumab-treated patients than those who received placebo (23.3% versus 62.2%; Δ − 38.9% [95% CI, − 56.0 to − 17.3], *P* = .0004).^67^

#### Patient-Reported Outcomes and HRQoL

Vedolizumab treatment is associated with improvements in patient-reported outcome measures and HRQoL. In the GEMINI LTS study, patient-reported HRQoL outcomes were reported using 3 validated measures that included Inflammatory Bowel Disease Questionnaire scores. Vedolizumab use was associated with improvements in all measures of HRQoL; these improvements were sustained up to week 400 of treatment.^40^ Similar improvements in HRQoL were observed in the phase 3 VERSIFY and VISIBLE 2 studies and in real-world observations.^41,42,68^

#### Safety Outcomes

In an integrated safety data analysis of 6 clinical trials in CD and ulcerative colitis, there was no increase in adverse events (AEs) or serious adverse events (SAEs), including gastrointestinal events and infections, compared with placebo in 2830 patients who had 4811 patient-years of exposure to vedolizumab.^69^ The exposure-adjusted incidence rate of any infection was lower in patients who received vedolizumab than in those who received placebo (63.5/100 patient-years vs 82.9/100 patient-years), and most infections were mild or moderate in severity.^69^ As shown by data from the GEMINI LTS study, the safety profile of vedolizumab in patients with CD was consistent over a period of 8 years. During this time, there were no unexpected safety concerns associated with vedolizumab use, including no increase in infections, infusion-related reactions, hepatic events, or cases of progressive multifocal leukoencephalopathy.^40^ When comparing malignancies reported in 1785 patients in GEMINI LTS who had at least 1 year of follow-up after vedolizumab initiation with rates in patients in a commercial database, the number of malignancies in those treated with vedolizumab was no higher than in the age- and sex-matched control population.^70^ In VISIBLE 2, overall safety findings were similar between patients who received vedolizumab SC and those who received placebo; 4.0% and 8.2% of patients who received vedolizumab SC and placebo, respectively, discontinued the study owing to AEs. In patients who received vedolizumab SC, 73.5% experienced an AE, compared with 76.1% in patients who received placebo. SAEs occurred in 8.4% and 10.4% of patients who received vedolizumab SC and placebo, respectively.^41^ The safety profile of vedolizumab SC in the VISIBLE 2 study was consistent with the known safety profile of vedolizumab IV as described in the GEMINI 2 study.^37^

These clinical trial findings are supported by data from the Vedolizumab Global Safety Database. Postmarketing surveillance data from more than 208 050 patient-years of exposure between launch (May 2014 in the EU and USA) and May 2018 demonstrated that the safety profile of vedolizumab was consistent with that observed in clinical trials; gastrointestinal events (identified using the “GI disorders” Medical Dictionary for Regulatory Activities version 21.0 system organ class) were the most frequently reported of all AEs in patients with CD (*n* = 6156 [16%]); these included exacerbation of CD, diarrhea, abdominal pain, nausea, vomiting, and rectal bleeding.^71^ Real-world data from the 24-month retrospective EVOLVE study of adults who received vedolizumab or anti-TNFα treatment in Canada, Greece, and the USA indicate that vedolizumab treatment in biologic-naive patients is associated with lower incidence rates of SAEs and serious infections than anti-TNFα treatment.^72,73^ A separate retrospective safety analysis of data from 1266 patients with CD included in the US VICTORY consortium registry found a lower rate of noninfectious SAEs in patients treated with vedolizumab than in those who received an anti-TNFα treatment (OR, 0.072; 95% CI, 0.012 to 0.242) but no difference in the rate of serious infections (OR, 1.183; 95% CI, 0.786 to 1.795).^74^ Overall, vedolizumab has a well-established safety profile, with available evidence suggesting safety event rates with vedolizumab are comparable to or lower than those with anti-TNFα treatments.

The safety profile of vedolizumab makes it an excellent candidate to form the backbone of combination therapy in CD. Although they are effective for many patients, advanced therapies, including biologics, do not work uniformly well when given as monotherapies, and some patients experience a primary nonresponse or secondary loss of response to treatment.^75,76^ Combining advanced therapies in patients with CD has been proposed to overcome this efficacy ceiling.^77^ The prospective EXPLORER study showed that combination therapy with vedolizumab, adalimumab, and methotrexate had a higher probability of producing better rates of endoscopic remission in patients with moderate to severe CD who were at risk of disease-related complications than monotherapy or placebo. Crucially, in EXPLORER, there were no new safety signals associated with the combination therapy regimen.^78^

### Vedolizumab Achieves Mucosal Healing in Patients With CD

Mucosal healing, also termed endoscopic remission, is associated with better long-term patient outcomes and is considered among the best measures of a treatment’s ability to modify the natural history of CD. Although it was not included as an endpoint in the pivotal GEMINI 2 study, other clinical and real-world studies have demonstrated the ability of vedolizumab to achieve mucosal healing (**Table 2**).^42^ The phase 3b, open-label VERSIFY study was the first large prospective study to evaluate endoscopic, radiologic, and histologic healing with vedolizumab in patients with active CD. At week 26, 11.9% of patients had achieved the primary endpoint of endoscopic remission (defined as an SES-CD ≤ 4). In a 52-week substudy population (*n* = 56), 17.9%, 16.1%, and 17.9% had endoscopic remission at weeks 14, 26, and 52, respectively.^42^ Like data for clinical outcomes, endoscopic remission rates were higher in those who were anti-TNFα treatment-naive than in those who had received prior anti-TNFα treatment. In the primary study population (*n* = 101), 54.5% had prior anti-TNFα treatment failure. At week 26, endoscopic remission rates were 19.6% for those who were anti-TNFα treatment-naive and only 5.5% for those whose prior anti-TNFα treatment had failed. Endoscopic remission rates were also higher in those who had moderate disease at baseline than in those with severe disease.^42^ In VERSIFY, a post hoc analysis also demonstrated that vedolizumab reduced the number of patients with evidence of MRE-detected transmural inflammation at weeks 26 and 52 compared with baseline.^81^

**Table 2.** Endoscopic outcomes in clinical trials and real-world studies of vedolizumab in patients with CD.

Study name	Endpoint/Outcome (definition)	Time point	Rate
**Clinical trials**			
VERSIFY^42^	Endoscopic response (SES-CD ≥ 50% decrease)	Week 14	33.7%
		Week 26	24.8%
		Week 52	53.6%^a^
	Endoscopic remission (SES-CD ≤ 4)	Week 14	16.8%
		Week 26	11.9%
		Week 52	17.9%^a^
	Complete mucosal healing (absence of any ulcers, including aphthae)	Week 14	11.9%
		Week 26	14.9%
		Week 52	17.9%^a^
LOVE-CD^43^	Endoscopic response (SES-CD ≥ 50% decrease)	Week 26	40%
		Week 52	45%
	Endoscopic remission (SES-CD < 4)	Week 26	33%
		Week 52	36%
**Real-world data**			
OBSERV-IBD^45^	Mucosal healing (absence of any ulcer)	Weeks 30-54	29.8%
US VICTORY consortium registry analysis^47^	Mucosal healing (absence of ulcers and/or erosions)	Month 6	21%
		Month 12	67%
Christensen 2018^56^	Endoscopic improvement (SES-CD > 50% decrease)	Month 6	40%
	Mucosal healing (SES-CD < 3)	Month 6	30%
Schreiber 2018^48^	Mucosal healing (Meta-analysis endpoint definitions include absence of mucosal ulcers and/or erosions; CDEIS < 3)	Month 6	19%-30%
		Month 12	6%-63%
	Endoscopic improvement (Meta-analysis endpoint definitions include CDEIS > 50% mean change; endoscopist’s final impression of visible CD activity compared with baseline)	Week 16	53%
		Week 52	50%
Dreesen 2018^79^	Mucosal healing (complete absence of ulcerations)	Week 22	23%^b^
Kotze 2018^80^	Endoscopic mucosal healing (complete mucosal normalization)	Month 3	22.2%
		Month 6	33.3%
		Month 12	25.9%
Kopylov 2018^57^	Endoscopic response (clear endoscopic improvement but with detectable ulcerations)	Week 26	63.7%
	Mucosal healing (absence of ulcerations in patients who had ulcerations at baseline ileocolonoscopy)	Week 26	45.5%
Plevris 2019^58^	Mucosal healing (absence of mucosal ulceration/erosions on ileocolonoscopy and complete tapering of steroids; MRI assessment or capsule endoscopy when ileocolonoscopy was not possible, defined according to local-site HCP)	Month 6	10.1%
		Month 12	38.9%
Faleck 2019^59^	Endoscopic remission (absence of ulcers and/or erosions)	Month 6	Early-stage CD^c^: 29%; late-stage CD^d^: 13%
Perin 2019^60^	Endoscopic remission (complete absence of ulcers)	Follow-up (mean 7.66 months)	36.11%
Hanžel 2019^61^	Endoscopic remission (SES-CD < 4 with the absence of any mucosal alteration)	Week 54	29%

^a^Data from sub-study population at week 52 (n = 56 patients).

^b^In 13 patients, the maintenance treatment schedule was intensified, implying an increase in dosing frequency from the label to every 4 weeks.

^c^Early-stage CD was defined as disease duration ≤ 2 years.

^d^Late-stage CD was defined as disease duration > 2 years. Vedolizumab 300 mg was administered intravenously every 8 weeks after induction unless otherwise stated.

Abbreviations: CD, Crohn’s disease; CDEIS, Crohn’s Disease Endoscopic Index of Severity; HCP, health care professional; IBD, inflammatory bowel disease; MRI, magnetic resonance imaging; SES-CD, Simple Endoscopic Score for Crohn’s Disease.

In the LOVE-CD study, endoscopic remission (SES-CD < 4) was observed in 33% and 36% of patients at weeks 26 and 52, respectively.^43^ Similar to the results of VERSIFY, higher rates of endoscopic remission were observed at weeks 26 and 52 in patients who were anti-TNFα treatment-naive than in those who had previously received an anti-TNFα treatment. A meta-analysis of the real-world effectiveness of vedolizumab identified 12 studies that reported mucosal healing data and 4 studies that reported endoscopic improvement.^48^ At 12 months, mucosal healing rates for patients with CD who were treated with vedolizumab ranged from 6% to 63%, and 50% of patients had endoscopic improvement.^48^ Of 212 patients included in the real-world VICTORY consortium registry, 121 received vedolizumab and were objectively assessed for mucosal healing by endoscopy. Of these, 67% had mucosal healing after 12 months of maintenance treatment with a median time to achieving healing of 33 weeks.^47^ Patients who had moderate disease severity at baseline were more likely than those with severe disease to have mucosal healing (HR, 0.54; 95% CI, 0.31 to 0.93). Deep remission (clinical remission and mucosal healing) was observed in 29% of patients at 12 months after treatment initiation.^47^

### Prior Anti-TNFα Treatment Diminishes the Effectiveness of Vedolizumab

There is considerable evidence from clinical trials and real-world studies that prior exposure to anti-TNFα treatment has a negative impact on the efficacy of some biologics, including vedolizumab, in patients with CD.^82^ Therefore, choosing the correct biologic treatment sequence may be important to achieving optimal treatment outcomes. In the GEMINI 3 study, the differences in clinical remission rate between patients who received vedolizumab and those who received placebo were 3.0% (95% CI, − 4.5% to 10.5%; *P* = .433) and 14.4% (95% CI, 5.7% to 23.1%; *P* = .001) at weeks 6 and 10, respectively, in patients who had prior anti-TNFα treatment failure. By comparison, in patients who were anti-TNFα treatment-naive, the differences were 19.2% (95% CI, 3.3% to 35.0%) and 19.1% (95% CI, 2.4% to 35.8%) at weeks 6 and 10, respectively.^38^ In a post hoc analysis of data from the GEMINI 2 and GEMINI 3 studies, when stratified by prior anti-TNFα treatment, the difference in clinical remission rate between vedolizumab and placebo at week 6 was 4.1% for patients with prior anti-TNFα treatment failure compared with 12.6% for patients who were anti-TNFα treatment-naive.^83^ At week 52, the differences in clinical remission rates between vedolizumab and placebo were 22.1% for anti-TNFα treatment-naive patients and 14.9% for patients with prior anti-TNFα treatment failure.^83^

Data from the VICTORY consortium demonstrated that patients with CD who had prior anti-TNFα treatment exposure were less likely to be in clinical remission or have mucosal healing with vedolizumab treatment than were those who were anti-TNFα treatment-naive (HR, 0.4 [95% CI, 0.20 to 0.81] and HR, 0.29 [95% CI, 0.12 to 0.73], respectively). Those who were anti-TNFα treatment-naive also had higher rates of clinical response and corticosteroid-free clinical remission and a lower rate of progression to surgery with vedolizumab than those who had prior anti-TNFα treatment exposure.^47^

LOVE-CD enrolled patients with active CD, 88% of whom had previously been exposed to anti-TNFα treatment. In the overall cohort, 33% and 36% of patients achieved endoscopic remission at weeks 26 and 52, respectively.^43^ When stratifying by prior anti-TNFα treatment, a clear difference in response to vedolizumab treatment was observed, although the number of anti-TNFα treatment-naive patients was small. At week 52, 62% of those who were anti-TNFα treatment-naive had endoscopic remission with vedolizumab versus 33% of those who had prior exposure to anti-TNFα treatment.^43^ In a real-world retrospective cohort study in Europe that included 184 anti-TNFα treatment-naive patients, the effectiveness of vedolizumab was higher than in studies that included patients previously exposed to anti-TNFα treatments. By week 14, 82% of patients had a clinical response, 64% were in clinical remission, and 52% were in corticosteroid-free clinical remission. At the last follow-up (week 44), these rates were 77%, 69%, and 60%, respectively.^57^

Whereas evidence suggests that the effectiveness of vedolizumab is reduced when it is used following prior anti-TNFα treatment, the converse does not appear to be the case. EVOLVE was a multicenter retrospective study that included 491 patients with CD who were treated with vedolizumab or an anti-TNFα treatment as a first-line biologic. Although the cohorts were too small to provide a definitive conclusion, data from patients with CD in EVOLVE showed that rates of clinical response and clinical remission were higher at 3 months and 6 months, respectively, in patients receiving a second-line anti-TNFα treatment after discontinuing vedolizumab than they were at 3 months and 6 months in patients receiving a first-line anti-TNFα treatment.^72^ This suggests that prior vedolizumab exposure does not affect the effectiveness of the subsequent anti-TNFα treatment.^72,82,84^ Overall, these data support the use of vedolizumab as a first-line biologic before the use of anti-TNFα treatments in patients with CD to maximize the therapeutic benefits of both drugs. Safety data also support this recommendation, with data from EVOLVE demonstrating that incidences of SAEs and serious infections were lower in patients who received first-line vedolizumab than in those who received a first-line anti-TNFα treatment: HR (95% CI), 0.49 (0.28 to 0.86) and 0.26 (0.08 to 0.87), respectively.^72^

For other advanced IBD therapies, including IL-23 inhibitors and JAK inhibitors, observational and clinical trial data on their use in sequence with vedolizumab remains limited. There is an unmet need for further study of sequences involving these treatments to adequately determine whether it has an impact on efficacy, as is seen with anti-TNFα treatments.

### Vedolizumab Is Most Effective When Used in Patients With Early CD

Evidence suggests that using anti-TNFα treatments early in the disease course can result in better outcomes in patients with CD.^85,86^ Consequently, the American Gastroenterological Association (AGA) guidelines recommend early biologic therapy in patients with moderate to severe CD.^87^ There is good evidence that outcomes with vedolizumab are similarly affected by disease duration, supporting its use early in the disease course without the need to wait for conventional therapy failure.^37,42,43,59,88–91^

In the VERSIFY study, although the overall number of patients enrolled was relatively small, the duration of CD was a good predictor of endoscopic remission. In the primary study population, 37.5% of patients with a disease duration of less than 1 year were in endoscopic remission at week 26 compared with only 10.5% of patients with a disease duration of between 3 years and 7 years.^42^ Analysis of endoscopic remission rates across disease duration groups in LOVE-CD revealed that at week 26, 36% of patients with 5 years or less since diagnosis had endoscopic remission versus 27% with 9 to 15.5 years and 30% with greater than 15.5 years. At week 52, the proportion with endoscopic remission increased to 46% in those with a disease duration of 5 years or less but remained at less than 27% in the longer disease duration groups.^43^ A further analysis of data from LOVE-CD stratified patients by early (< 2 years since diagnosis and treated only with corticosteroids and immunomodulators) and late (> 2 years since diagnosis and treated with corticosteroids plus immunomodulators and/or an anti-TNFα treatment) disease. With a strict composite endpoint of combined clinical (CDAI ≤ 150) and endoscopic (SES-CD < 4) remission at both week 26 and 52, 31.4% of patients with early disease reached the endpoint versus 8.6% of those with late disease (*P* = .001).^91^

Patients in the VICTORY consortium who had a disease duration of 2 years or less were significantly more likely to have clinical remission (38% vs 23%), corticosteroid-free clinical remission (43% vs 14%), or endoscopic remission (29% vs 13%) after 6 months of vedolizumab treatment than those with a disease duration of more than 2 years.^59^ Furthermore, a post hoc analysis of the GEMINI 2 study found that disease durations of less than 2 years and less than 5 years were good predictors of achieving a composite endpoint of an abdominal pain score of 1 or less and a stool frequency score of 3 or less at week 2^89^ and a subgroup analysis of data from GEMINI 2 found that the differences in clinical remission rate between vedolizumab and placebo groups were higher in patients with a disease duration of less than 7 years (≥ 1 year to < 3 years: 27.2%; ≥ 3 years to < 7 years: 21.8%) than in those with longer duration disease (≥ 7 years: 10.8%).^37^

Overall, as has been observed with all advanced therapies used to treat CD, evidence suggests that therapeutic benefit is maximized if vedolizumab is initiated as a first-line biologic early in the disease course rather than waiting until after other treatments, including anti-TNFα treatments, have failed.

### Onset of Action of Vedolizumab in Patients With CD

There is a misperception that treatments that target cellular mechanisms are slower to act than targeted cytokine treatment strategies. In fact, one of the principal mechanisms of corticosteroids is thought to be a prominent lymphocytic effect. This misperception has contributed to the idea that vedolizumab is slow to act in patients with CD, a perception that may have arisen partly from the week 6 data from the GEMINI 2 and 3 studies. In GEMINI 2, the clinical response rate with vedolizumab was not significantly different to placebo at week 6 (31.4% vs 25.7%, *P* = .23) but was improved by week 52 (43.5% vs 30.1%, *P* = .01). In GEMINI 3, a similar pattern was observed, with no significant difference in clinical remission between vedolizumab and placebo at week 6, but followed by an improvement by week 10.^38^ However, prior exposure to an anti-TNFα treatment affects the effectiveness of vedolizumab and may delay a symptomatic response in patients.^47^ In GEMINI 2, approximately 50% of patients had prior exposure to anti-TNFα treatment, and in GEMINI 3, the primary endpoint data were from a cohort who all had prior anti-TNFα treatment failure.^37,38^ Indeed, in GEMINI 3, the difference in clinical remission between vedolizumab and placebo at week 6 was greater in those who were anti-TNFα treatment-naive (Δ: 19.2%; 95% CI, 3.3 to 35.0) than in those who had received prior anti-TNFα treatment (Δ: 3.0%; 95% CI, − 4.5 to 10.5).^38^

Overall, for many patients, particularly those who are anti-TNFα treatment-naive, available evidence suggests that vedolizumab begins to act within 6 weeks. A meta-analysis of real‑world effectiveness of vedolizumab conducted by Schreiber and colleagues found a combined clinical response rate in a total of 20 eligible studies of 56% (95% CI, 46% to 65%) at week 6 of treatment; the combined clinical remission rate in a total of 18 eligible studies was 24% (95% CI, 20% to 27%) at week 6.^48^ These data suggest that most patients treated with vedolizumab have a response by week 6 and that 1 in 4 have remission by that time. Of 644 patients who received induction doses of vedolizumab at weeks 0 and 2 in the VISIBLE 2 study, 412 patients (64%) had a clinical response at week 6; over 50% of patients with a response at week 6 had previous anti-TNFα treatment exposure.^41,92^

Looking at data before week 6, a post hoc analysis of more than 700 patients in the GEMINI 2 and GEMINI 3 studies found that patients with moderate to severe CD who received vedolizumab had a greater reduction in combined abdominal pain and loose stool frequency score at weeks 2, 4, and 6 than patients who received placebo: difference adjusted percentage change (vedolizumab − placebo) of − 7.0% (95% CI, − 11.0% to − 2.9%), − 8.6% (95% CI, − 13.2% to − 4.0%), and − 10.4% (95% CI, − 15.4% to − 5.4%), respectively.^89^ In the VISIBLE 2 study, a weekly decrease in combined abdominal pain and stool frequency, as well as in patient-reported outcomes-2 scores, were observed between baseline and week 6, with 50% of the reduction occurring in the first 2 weeks.^93^ Additionally, the reductions reported in both the GEMINI and VISIBLE studies were greater for patients who were anti-TNFα treatment-naive.^89,93^ The perception that vedolizumab is generally slow to act in patients with CD is not supported by the evidence. When used in the appropriate patient population, particularly those who are anti‑TNFα treatment-naive, vedolizumab can act quickly, with relief of symptoms occurring in less than 2 weeks.

### Disease Location May Impact on the Effectiveness of Vedolizumab in Patients with CD

Disease location is a prognostic factor for disease progression and complications in CD, with the presence of ileal or ileocolonic disease typically predictive of a more complicated disease.^26,27^ Several clinical and real-world studies have reported the impact of disease location on the effectiveness of vedolizumab.^42,43,74,94–96^

A post hoc analysis of data from GEMINI 2 found similar rates of clinical response, clinical remission, and corticosteroid-free clinical remission at week 52 in patients with ileal, colonic, or ileocolonic disease.^94^ This was complemented by findings of a real-world retrospective study that showed disease location, according to the Montreal classification, did not influence the rate of corticosteroid-free remission with vedolizumab at week 24 in patients with CD; except for baseline active perianal disease which was associated with lower probability of corticosteroid-free remission at week 24.^95^ In VERSIFY, complete mucosal healing (defined as the absence of any ulcers, including aphthae) with vedolizumab was higher in the colon than in the ileum at weeks 14, 26, and 52; at all time points, rates for the transverse colon were approximately double those for the ileum.^42^ Initial findings from LOVE-CD showed a similar pattern to results reported in VERSIFY, with the proportion of patients in endoscopic remission at week 26 being higher in those with disease in the transverse colon than in those with disease in the ileum (71% vs 47%), although the difference was not statistically significant.^43^ However, a more recent analysis of data from LOVE-CD, which stratified patients by Montreal classification, did not find differences in endoscopic remission at week 52 across groups with L1 (28.6%), L2 (38.5%), and L3 (31.5%) disease.^96^

### Vedolizumab Does Not Appear to Exacerbate Extraintestinal Manifestations in Patients With CD

Extraintestinal manifestations (EIMs) affect up to 50% of patients with IBD, can occur in up to 24% of patients before the onset of intestinal symptoms, and can have a marked impact on patients’ quality of life.^9,97^ A misconception is that successful treatment of intestinal inflammation will also produce satisfactory control of EIMs. Although anti-TNFα treatments have been shown to be successful in treating some EIMs, highlighting the role of tumor necrosis factor (TNF) in EIM pathophysiology, data on the effectiveness of vedolizumab in treating EIMs is less clear. Nonetheless, an impression exists that vedolizumab may not be effective in treating EIMs or may even exacerbate them. This may be because vedolizumab is considered gut-selective and is not expected to have systemic effects. The pathophysiological mechanisms underlying EIMs are complex and varied, and it is difficult to make predictions about the effectiveness of vedolizumab on this basis. A review of the relationship between vedolizumab and EIMs concluded that there is evidence in support of the effectiveness of vedolizumab in managing EIMs associated with active luminal disease. It was also suggested that apparent worsening of EIMs with vedolizumab may not be causal and could be “consistent with paradoxical inflammatory manifestations or the withdrawal of TNF antagonists and corticosteroids.”^97^

### Guidelines for the Use of Vedolizumab in Patients With CD

Based on available evidence, the AGA,^87^ the American College of Gastroenterology (ACG),^98^ the European Crohn’s and Colitis Organisation (ECCO),^99,100^ and the British Society of Gastroenterology (BSG)^101^ have created best practice guidance on the management of CD and the use of biologics, including vedolizumab. The current recommendations for vedolizumab are summarized in **Table 3**. Updated AGA and BSG guidelines were expected in 2024 but were not available at the time of writing.

**Table 3.** Clinical guidelines for the treatment of patients with CD with vedolizumab.

Guidelines	Recommendations
2024 ECCO Guidelines on Medical Treatment of CD^100^	**•**Recommended for induction therapy in patients with moderate to severe CD (Statement 12.1; strong recommendation, moderate-quality evidence)^a^ **•**Recommended for maintenance therapy in patients with moderate to severe CD (Statement 12.2; strong recommendation, moderate-quality evidence)^a^
ECCO Evidence-based Consensus on CD Diagnosis and Medical Management^99^	•Vedolizumab is an appropriate alternative in patients with moderately or severely active localized ileocecal CD who are refractory to steroids and/or anti-TNF therapy (Statements 5C and 5D; evidence level 1)^b^ •Vedolizumab is an appropriate alternative in patients with active colonic CD who are refractory to steroids and/or anti-TNF therapy (Statement 5E; evidence level 1)^b^ •Vedolizumab is appropriate for maintenance treatment in patients who have achieved remission with vedolizumab (Statement 6F; evidence level 1)^b^
2021 AGA Clinical Practice Guidelines^87^	**•**Suggested over no treatment for the induction and maintenance of remission in adult outpatients with moderate to severe CD (Recommendation 1B; conditional recommendation, low-quality evidence for induction, moderate-certainty evidence for maintenance)^c^ **•**Suggested for the induction of remission over certolizumab pegol in adult outpatients with moderate to severe CD who are naive to biologics (Recommendation 2A; conditional recommendation,^d^ low‑certainty evidence)^c^ **•**Suggested for the induction of remission over no treatment in adult outpatients with moderate to severe CD who have never responded to anti-TNFα therapy (Recommendation 2B; conditional recommendation,^d^ low‑certainty evidence)^c^ **•**Suggested for the induction of remission over no treatment in adult outpatients with moderate to severe CD who have previously responded to infliximab (Recommendation 2C; conditional recommendation,^d^ low‑certainty evidence)^c^
2018 ACG Guidelines^98^	**•**For patients with moderately to severely active CD and objective evidence of active disease, vedolizumab with or without an immunomodulator is more effective than placebo and should be considered to be used for induction of symptomatic remission (Recommendation 27; strong recommendation,^e^ high level of confidence)^f^ **•**Recommended for maintenance of remission in patients with CD who have achieved remission with vedolizumab (Recommendation 51; conditional recommendation,^e^ moderate level of evidence)^f^
2019 BSG Guidelines^101^	•Recommend that moderate to severely active uncomplicated luminal CD should be treated initially with systematic corticosteroids (Statement 37; strong recommendation,^g^ high-quality evidence)^h^, but suggest that those with extensive disease or other poor prognostic features should be considered for early introduction of biologic therapy (Statement 37; weak recommendation,^g^ moderate-quality evidence)^h^ •Recommend that patients refractory to immunomodulator therapy despite dose optimization should be considered for biologic therapy. Choice between anti-TNFα treatment, ustekinumab, and vedolizumab should be made on an individual basis, considering patient preference, cost, likely adherence, safety data, and speed of response to the drug (Statement 43; strong recommendation,^g^ very-low-quality evidence)^h^ •Recommend that in CD, vedolizumab can be used in both anti-TNF–naive patients and in those in which anti-TNFα treatment fails. Choice of treatment in biologic-naive patients should be individualized (Statement 46; for induction therapy: strong recommendation,^g^ moderate-quality evidence;^h^ for maintenance therapy: strong recommendation,^g^ high-quality evidence)^h^

^a^Moderate-quality evidence=further research may change the Guideline Committee’s confidence in the effect estimates.

^b^Evidence levels used according to the Oxford Centre for Evidence-Based Medicine (https://www.cebm.ox.ac.uk/resources/levels-of-evidence/ocebm-levels-of-evidence).

^c^Moderate-certainty evidence=the true effect is likely to be close to the estimate of effect, but there is a probability that it is substantially different; low-certainty evidence=the true effect may be substantially different from the estimate of the effect.

^d^Conditional recommendation=different choices would be appropriate for different patients.

^e^Strong recommendation=the desirable effect(s) clearly outweighs the undesirable effect(s); conditional recommendation=there is uncertainty about the trade-offs.

^f^High level of evidence=further research was unlikely to change the Guideline Committee’s confidence in the effect estimate; moderate level of evidence=further research would be likely to have an impact on the Guideline Committee’s confidence in the effect estimate.

^g^Strong recommendation=benefits clearly outweigh risks and burdens; weak recommendation=benefits, risks, and burdens are conditional, closely balanced, or uncertain.

^h^High-quality evidence=further research is unlikely to change confidence in the estimate of the effect; moderate-quality evidence=further research is likely to have an important impact on confidence in the estimate of the effect and may change the estimate; low-quality evidence=further research is very likely to have an important impact on confidence in the estimate of effect and is likely to change the estimate; very-low-quality evidence=any estimate of effect is uncertain.

Abbreviations: ACG, American College of Gastroenterology; AGA, American Gastroenterological Association; anti-TNF, anti-tumor necrosis factor; BSG, British Society of Gastroenterology; CD, Crohn’s disease; ECCO, European Crohn’s and Colitis Organisation.

The guidelines of these organizations were created using broadly similar processes involving the development of clinically relevant questions using the Population, Intervention, Comparator, Outcomes format followed by an assessment of available evidence in the literature and development of a set of consensus recommendations by expert panels using the Grading of Recommendations Assessment, Development and Evaluation (GRADE) approach. Using GRADE, the strength of each recommendation is assessed as “strong” or “weak/conditional” based on the quality of the supporting evidence, which is typically ranked from “very strong” (further research would be unlikely to change the confidence of the estimate of the effect) through “moderate,” “weak,” and “very weak” (any estimate of the effect is very uncertain).^87,98,100,101^

There is a broad consensus across guidelines regarding the use of vedolizumab in patients with CD. Although the strength of the recommendation varies, all guidelines recommend vedolizumab for induction of remission in patients with moderate to severe CD whose disease previously did not respond to at least 1 anti-TNFα treatment. As per their guidelines current as of August 2024, the AGA and BSG also recommend the use of vedolizumab for induction in patients who are biologic-naive.^87,101^ Moreover, BSG guidelines specify that for patients with extensive disease or other poor prognostic features, early introduction of biologic treatment should be considered.^101^ For maintenance treatment, all guidelines recommend the use of vedolizumab in patients with moderate to severe CD who have achieved clinical remission in response to induction therapy, including with vedolizumab. These treatment guidelines relate to the treatment of adults and may not be suitable for specific populations such as pregnant women or pediatric patients. In pregnant women, the Pregnancy in Inflammatory Bowel Disease and Neonatal Outcomes (PIANO) study has been ongoing since 2009. In data up to November 2022, 66 women had been exposed to vedolizumab during pregnancy without any observed increased risk in adverse pregnancy or neonatal outcomes versus non-exposed women.^102^ Although safety data for use in pregnant women remains limited, available data suggests that vedolizumab can be used without additional concerns regarding pregnancy outcomes. Finally, these guidelines cover adult patients with CD. Vedolizumab is not currently approved for use in pediatric patients, although phase 3 studies are ongoing to establish the efficacy and safety in this population.

### Patient Expectations and Shared-Decision Making in CD

Patients with IBD expect to be well informed about their disease and involved in the treatment decision-making process. A systematic review of studies that evaluated patient perspectives and preferences identified efficacy, safety, and convenience as the most important treatment expectations for people with CD.^103^ Although some studies suggested that patients with CD are prepared to balance rare risks (eg, infection) against improved rates of remission, the side effect profile of medications is consistently important to patient decision making, as is the route of administration (eg, IV vs SC).^103^ In a discrete choice experiment, most patients with CD prioritized maintaining remission as the most important factor when choosing a treatment. However, there were also more risk-averse groups of patients who favored vedolizumab over other treatments owing to its gut-selective mechanism of action and lower risk of systemic toxicity.^104^ Identifying individual patients’ preferences through shared treatment decision-making and involving patients early in the decision-making process is important to providing optimal care and avoiding misalignment between patient and physician expectations.

## Conclusion

**Figure 2.**
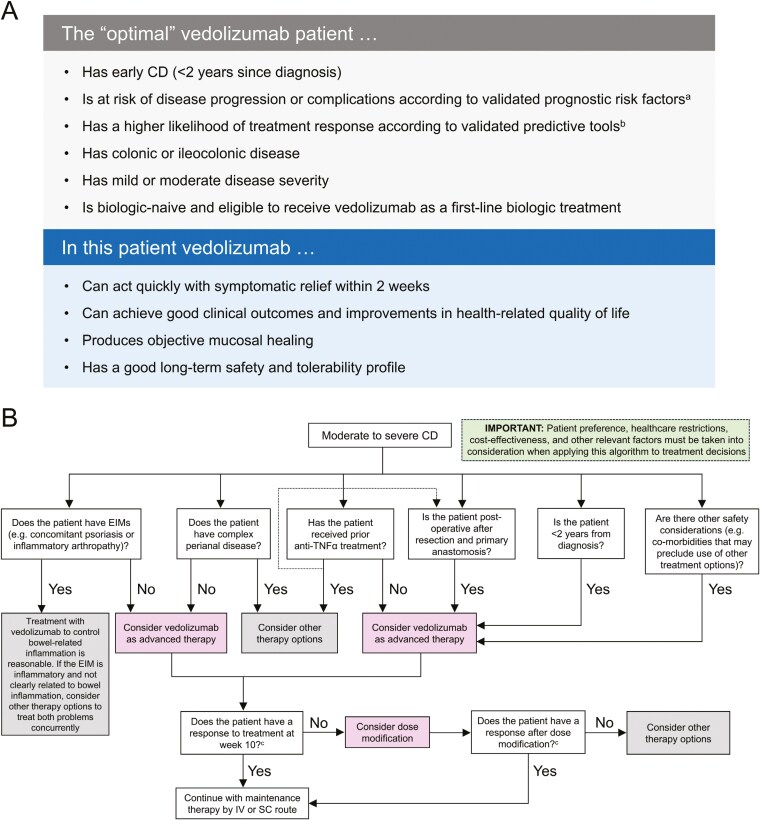
Characterization of the type of patient with CD for whom vedolizumab treatment is expected to be of optimal therapeutic benefit (A) and a vedolizumab treatment algorithm for people with CD (B). ^a^For example, those proposed by the American Gastroenterological Association.^27^^b^For example, vedolizumab clinical-decision support tools.^28,105^^c^As determined by an appropriate available clinical, endoscopic, biomarker, or other (eg, intestinal ultrasound/MRE) assessment. Abbreviations: CD, Crohn’s disease; EIM, extraintestinal manifestation; IV, intravenous; SC, subcutaneous; TNF, tumor necrosis factor.

Although vedolizumab has benefits for many patients, data suggest that optimal benefits will be achieved in patients who have early disease (≤ 2 years since diagnosis); are at risk of disease progression and complications according to validated prognostic risk factors; have mild or moderate colonic or ileocolonic disease; and are biologic-naive and eligible to receive vedolizumab as a first-line biologic (**Figure 2**). For similar reasons, it is a reasonable option to be used for the prevention of recurrence after a surgical resection and primary anastomosis. The relative lack of head-to-head comparative data with other therapies in patients with CD has made it historically challenging to determine when vedolizumab should be used and for which patients. When considering the use of vedolizumab for the treatment of patients with CD, the treatment algorithm we present in Figure 2 can act as a useful starting point, although care should be taken to also consider patient preference, healthcare restrictions, cost-effectiveness, and other relevant individual patient factors when making treatment decisions. Overall, vedolizumab acts quickly in most patients, producing symptomatic relief in up to 2 weeks, achieves positive clinical outcomes and improvements in HRQoL that are associated with objective mucosal healing, and has a good long-term safety and tolerability profile.
